# Plastic Surgeons’ Attitudes and Understanding of Body Dysmorphic Disorder

**DOI:** 10.7759/cureus.72630

**Published:** 2024-10-29

**Authors:** Amal Y Alhazmi, Sofana N Faqih, Bayader S Alsalem, Moayyad S Alsalem, Hatem Alnoman

**Affiliations:** 1 Department of Psychiatry, King Abdullah International Medical Research Center, Jeddah, SAU; 2 Department of Medicine, Psychiatry Section, King Abdulaziz Medical City - National Guard Health Affairs, Jeddah, SAU; 3 Department of Psychiatry, Eradah Mental Health Complex, Jeddah, SAU; 4 Department of Internal Medicine, King Faisal Hospital, Makkah, SAU; 5 Department of Internal Medicine, Psychiatry Section, King Saud bin Abdulaziz University for Health Sciences, King Abdulaziz Medical City - National Guard Health Affairs, Jeddah, SAU; 6 Department of Surgery, Plastic Surgery Section, King Abdulaziz Medical City - National Guard Health Affairs, Jeddah, SAU

**Keywords:** aesthetic treatment, body dysmorphic disorder, contraindication, familiarity, plastic surgeons, saudi arabia

## Abstract

Introduction

Body dysmorphic disorder (BDD) involves an intense preoccupation with perceived or minor defects in physical appearance. Patients with BDD often experience dissatisfaction across various domains, including mental and physical well-being, relationships, and role functioning.

Purpose

This study evaluated the awareness, knowledge, and attitudes of plastic surgeons in Saudi Arabia toward BDD.

Participants and methods

This was a cross-sectional study that included board-certified plastic surgeons in Saudi Arabia. The data was collected through a self-administered survey from July 2022 until May 2023. The total number of participants was 213. The survey includes questions about demographic data, familiarity with BDD, and participant’s attitudes toward BDD. The questionnaire was adapted from Bouman et al.’s study.

Results

Most of the participants were familiar with the clinical picture of BDD; 46.9% were reasonably familiar with the clinical picture of BDD, and 11.3% were totally familiar with the diagnosis. Furthermore, the most common symptoms frequently seen in patients with BDD were “dissatisfaction with previous cosmetic surgery” (68.5%) and “unusual/excessive requests for cosmetic surgery” (67.6%). However, only 21.1% of participants will consult a psychiatrist/psychologist about what to do before proceeding to a cosmetic procedure. Notably, the potential verbal and physical aggression encountered by our participants was 85% among patients with BDD.

Conclusion

This research demonstrated that plastic surgeons exhibit a high familiarity with BDD. Also, the findings of the current study could be valuable in shaping policies and providing recommendations to assist plastic surgeons and cosmetic treatment institutes in managing BDD patients. Subsequently, a significant proportion of our participants reported receiving threats from BDD patients. Therefore, this highlights the need to explore the risk of post-surgery violence in individuals with BDD.

## Introduction

Body dysmorphic disorder (BDD) is characterized by an excessive focus on perceived or minor flaws in physical appearance [1]. The disorder significantly impairs quality of life, affecting mental and physical health, relationships, and role functioning [2]. Despite its psychiatric nature, patients with BDD more commonly consult dermatologists or plastic surgeons rather than psychiatrists [3]. According to the Diagnostic and Statistical Manual of Mental Disorders, Fifth Edition (DSM-V), the prevalence among United States adults is 2.4%, with rates of 2.5% in females and 2.2% in males. [1]. However, these rates escalate to 11-13% among dermatology patients and 13%-15% of cosmetic surgery patients internationally. In the orthodontic and oral/maxillofacial surgery populations, the prevalence is 5% and 10%, respectively [1]. In Saudi Arabia, one study in Riyadh reported a 4.4% prevalence among medical students, while another in Jeddah found a 19.2% prevalence among plastic and oculoplastic surgery patients [4,5].

Although BDD patients frequently seek cosmetic treatments, satisfaction is generally low; 72% experienced no change post-procedure, and 16% worsened [6]. Dissatisfied patients may become litigious or violent toward their physicians [7]. The challenge for healthcare providers lies in managing BDD patients’ unrealistic expectations and persistent dissatisfaction [8]. A survey revealed that 60% of surgeons consider BDD a contraindication for aesthetic procedures [9].

Internationally, studies have assessed plastic surgeons’ awareness of BDD. In Europe, a 2015 survey among Dutch professionals found that 50.9% had long been familiar with BDD. However, only two-thirds reported encountering one to five BDD patients annually, lower than the expected prevalence [10]. In contrast, United States respondents reported encountering approximately 2% of BDD patients, aligning closely with prevalence rates [11].

Despite the critical need, we found only one study conducted in Saudi Arabia regarding plastic surgeons’ awareness of BDD. The study included 155 participants: 56 plastic surgeons, 98 dermatologists, and one otolaryngologist. More than half were familiar with BDD diagnostic criteria, though 12.3% were completely unfamiliar. Unlike dermatologists, over half of plastic surgeons will not tell the patient if the aesthetic procedure is unnecessary. Only 11% of participants would consider referral to psychiatry before proceeding to plastic surgery [12].

Cosmetic surgery is burgeoning in Saudi Arabia, and BDD patients more commonly consult cosmetic treatment providers than psychiatrists. Assessing the awareness of plastic surgeons about BDD is thus vital. This study aims to assess the awareness of BDD among plastic surgeons in Saudi Arabia. Additionally, to explore the plastic surgeons' attitudes and experiences with BDD and to investigate the association between years of experience, gender, and knowledge of BDD.

## Materials and methods

Participants

This cross-sectional study included all board-certified plastic surgeons working in private and public hospitals in Saudi Arabia. Data were collected via a self-administered survey from July 2022 to May 2023, with ethical approval from the institutional review board at the King Abdullah International Medical Research Center (KAIMRC), with reference number NRJ22J/084/03. Surgeons were approached by a digital mailing list provided by the Saudi Commission for Health Specialties. We did not provide any information regarding BDD before distributing the survey. A convenience sampling technique was used, and the sample size calculation was based on a population of 470 plastic surgeons, per Ministry of Health statistics, with a 95% confidence level and a 5% margin of error, resulting in an estimated sample size of n = 213.

Procedures

The survey comprised four sections: part I captured demographic data such as age, sex, years of experience, annual patient inflow, and nationality; part II assessed familiarity with BDD; part III gauged attitudes toward BDD; and part IV explored how participants manage patients with suspected BDD (see Appendix). The questionnaire was adapted from a study by Bouman et al. The questionnaire was tested in a pilot study. Feedback was obtained from experts to identify unclear questions and to verify the content and face validity, ensuring that the questions accurately aligned with the study's objectives. Around 50% of participants responded to the e-mail survey, and a follow-up reminder was sent two weeks after the initial survey distribution to improve response rates. No incentives were offered to the participants. 

Attitudes toward BDD were evaluated using a 12-item, five-point Likert scale questionnaire. Total scores ranged from 12 to 60, with higher scores indicating more positive attitudes toward BDD. Surgeons were categorized based on these scores: <50% as negative, 50%-75% as neutral, and >75% as positive.

Participation was voluntary, and withdrawal was permitted at any time. The survey’s introductory page included informed consent and the study’s aims. Data were collected in English, with stringent measures to ensure participant confidentiality and anonymity. All data were stored on a secure workplace research-team-accessible computer.

Pilot study

We performed a pilot study among 30 participants to determine the reliability and validity of the study questionnaire. The sample was selected randomly. The questionnaire to be measured comprised 12 attitude items, with a five-point Likert scale ranging from “Don’t agree at all” coded with one to “Totally agree” coded with five. We also measured the reliability of experience with a BDD questionnaire consisting of three items, with a four-point Likert scale category ranging from “Never” coded with one to “always” coded with four. The reliability test for the attitude questionnaire has a Cronbach alpha of 0.814 or 81.4%, indicating very good internal consistency, while the reliability test of the experience items has a Cronbach alpha of 0.707 or 70.7%, indicating good internal consistency. The overall reliability test of the study questionnaire consisting of 15 items has a Cronbach alpha of 0.761 or 76.1%, suggesting a good internal consistency. Thus, the questionnaire was valid to use in this study and did not require further changes. 

Statistical analysis

Data analysis was conducted using SPSS (IBM SPSS Statistics for Windows, IBM Corp., Version 26, Armonk, NY). Categorical variables were presented as numbers and percentages, while continuous variables were summarized using mean and standard deviation. Normality was assessed using the Shapiro-Wilk and Kolmogorov-Smirnov tests. Due to the non-normal distribution of attitude scores, nonparametric tests were employed. Associations between surgeons’ knowledge and demographic characteristics were analyzed using the chi-square test. Differences in attitudinal scores relative to socio-demographic characteristics were evaluated using the Mann-Whitney U test. p-values < 0.05 were considered statistically significant.

## Results

Demographics

This study involved 213 plastic surgeons. As detailed in Table 1, 34.7% (n = 74) were aged 41-50, and most were male (52.6%, n = 112) Saudi nationals (82.6%, n = 176) with 16-20 years of professional experience. Consultants comprised 52.1% (n = 111) of the sample, and 66.2% of the participants (n = 141)worked in private clinics. Moreover, 36.6% (n = 78) reported acquiring 351-500 new patients annually.

**Table 1:** Socio-demographic characteristics of plastic surgeons (n = 213) † Some surgeons have more than one employment setting.

Study data	n (%)
Age group	
25-30 years	28 (13.1%)
31-40 years	71 (33.3%)
41-50 years	74 (34.7%)
>50 years	40 (18.8%)
Gender	
Male	112 (52.6%)
Female	101 (47.4%)
Nationality	
Saudi	176 (82.6%)
Non-Saudi	37 (17.4%)
Years of experience	
<5 years	26 (12.2%)
5-10 years	42 (19.7%)
11-15 years	63 (29.6%)
16-20 years	82 (38.5%)
Current position	
Consultant	111 (52.1%)
Specialist	86 (40.4%)
Board certified physician	16 (07.5%)
Employment setting ^†^	
General hospital	125 (58.7%)
Private clinic	141 (66.2%)
Self-employed	37 (17.4%)
University hospital	29 (13.6%)
Number of new patients each year	
<200	15 (07.0%)
200-350	50 (23.5%)
351-500	78 (36.6%)
>500	70 (32.9%)

Knowledge

Table 2 reveals that BDD patients’ most frequently observed symptom was "dissatisfaction with prior cosmetic surgery" 68.5% (n = 146). Awareness of BDD diagnostic criteria was reported by 37.1% (n = 79). The majority of participants (93%, n = 198) demonstrated varying degrees of familiarity with the clinical presentation of BDD; 38% (n = 81) had definitively encountered BDD patients, and 41.8% (n = 89) reported seeing five to 10 patients with BDD over the last year.

**Table 2:** Assessment of knowledge toward BDD (n = 213) † Variable with multiple response answers. BDD, body dysmorphic disorder

Knowledge statement	n (%)
Please select the symptoms that you have most frequently encountered/seen in patients whom you suspected to have BDD ^†^	
"Dissatisfaction with previous cosmetic surgery"	146 (68.5%)
"Unusual or excessive requests for cosmetic surgery"	144 (67.6%)
"References to others taking special note of the perceived appearance flaw"	81 (38.0%)
"Excessive concern with, or distress over, or minor or nonexistent appearance flaws"	72 (33.8%)
"Belief that the procedure will transform patient’s life or solve all problems"	60 (28.2%)
Are you familiar with the diagnostic criteria for BDD?	
"I’m seeing these criteria now for the first time"	22 (10.3%)
"I’ve heard of these"	79 (37.1%)
"I’m slightly familiar with these"	72 (33.8%)
"I have been familiar with these for a long time"	40 (18.8%)
Are you familiar with the clinical picture of BDD	
"I’m not familiar with this"	15 (07.0%)
"I’m partly familiar with this"	74 (34.7%)
"I’m reasonably familiar with this"	100 (46.9%)
"I’m totally familiar with this"	24 (11.3%)
How did you acquire knowledge of BDD? ^†^	
No knowledge	13 (06.1%)
General Professional Literature	76 (35.7%)
Specific Literature on BDD	107 (50.2%)
Conferences or Lecture	103 (48.4%)
Colleagues	65 (30.5%)
Websites	30 (14.1%)
Did you encounter patients with BDD over the past year?	
"No, I didn’t encounter any"	07 (03.3%)
"Yes, by hindsight, I’ve probably encountered them"	98 (46.0%)
"Yes, by hindsight, I’ve certainly encountered them"	81 (38.0%)
"Yes, I’ve certainly encountered them"	27 (12.7%)
How many patients with BDD did you see last year?	
None	07 (03.3%)
One to five patients	78 (36.6%)
Five to 10 patients	89 (41.8%)
>10 patients	39 (18.3%)

Attitudes

**Figure 1:**
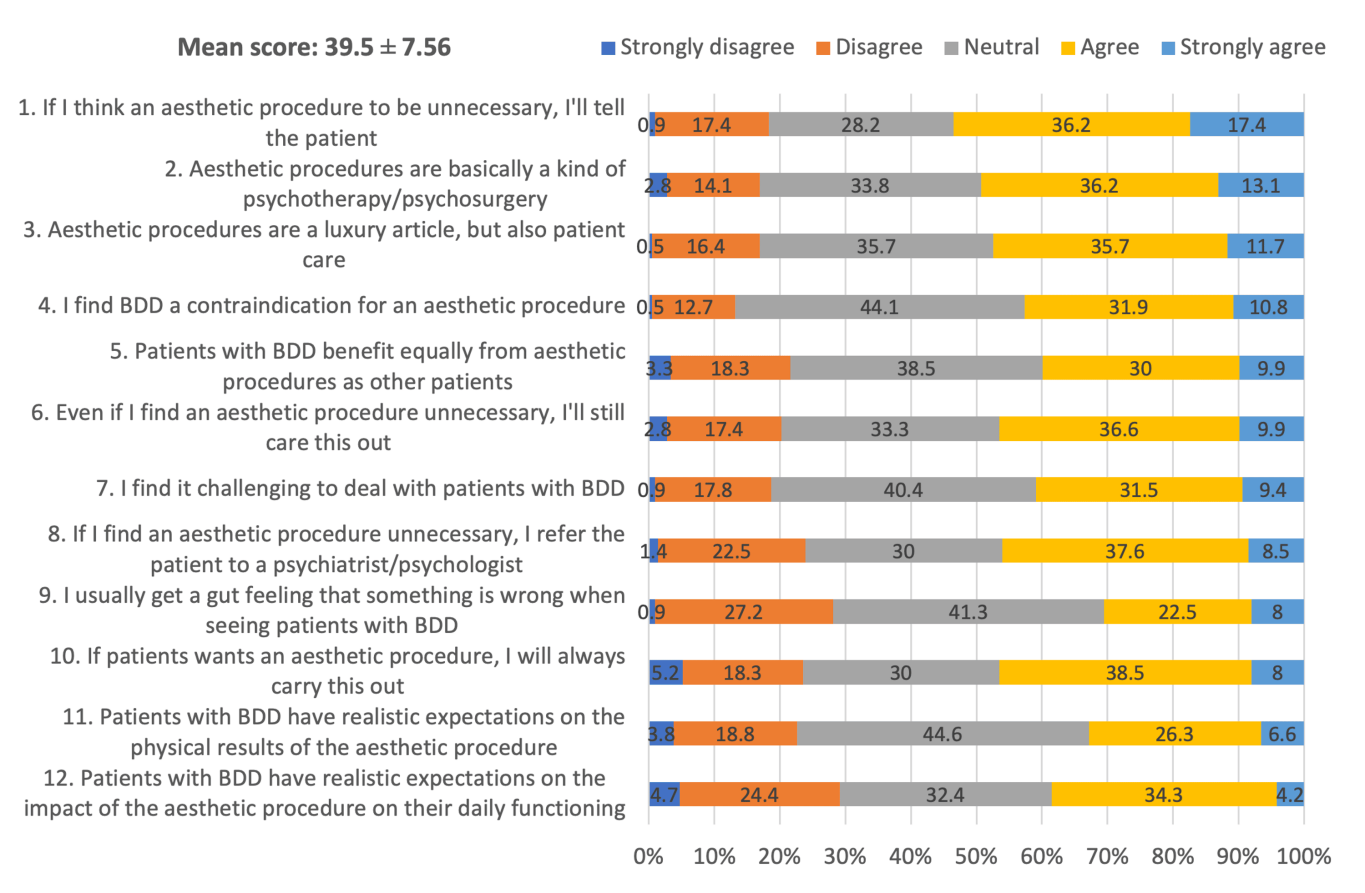
Assessment of attitude toward body dysmorphic disorder (BDD)

Figure 1 highlights the top three attitudes toward BDD. The most agreed-upon statements were "If I deem a procedure unnecessary, I will tell the patient" (strongly agree: 17.4%), followed by "Aesthetic procedures essentially serve as psychotherapy" (strongly agree: 13.1%), and "Aesthetic procedures, while luxurious, also provide patient care" (strongly agree: 11.7%). Surgeons’ attitudes were categorized into negative, neutral, and positive. Notably, 73.7% exhibited neutral attitudes, 17.4% positive, and 8.9% negative.

Table 3 indicates that only 8.5% (n = 18) routinely assess for BDD during intake interviews, and just 10.3% (n = 22) consistently steer the conversation toward body image issues; 8.5% (n = 18) reported disclosing a BDD diagnosis to a patient. The most common approach for suspected BDD patients was discussing the distorted body image 26.3% (n = 56). Additionally, 45.5% (n = 97) had often declined to treat a BDD patient, and 38.5% (n = 82) reported being physically threatened by a BDD patient.

**Table 3:** Assessment of experience with BDD (n = 213) † Variable with multiple response answers.

Statement	n (%)
Do you explore BDD or disturbed body image during the intake interview?	
Never	04 (01.9%)
Sometimes	124 (58.2%)
Often	67 (31.5%)
Always	18 (08.5%)
In patients with BDD do you shift the topic from the technical aspects of the procedure to the body image problem?	
No, body image is never part of the intake interview	12 (05.6%)
Sometimes	95 (44.6%)
Most of the time	84 (39.4%)
Always, because body image is a standard topic during the intake interview	22 (10.3%)
In case of BDD or disturbed body image, do you share this with your patient?	
No, I keep that to myself	12 (05.6%)
Sometimes	80 (37.6%)
Most of the time	103 (48.4%)
Yes, always	18 (08.5%)
What do you do when you recognize or suspect BDD in a patient?	
I don’t address this	05 (02.3%)
I approach such a patient no different from other patients	14 (06.6%)
I share my impression of this patient’s appearance	39 (18.3%)
I talk about the patient’s disturbed body image	56 (26.3%)
First, I consult a psychiatrist/psychologist about what to do	45 (21.1%)
I refer the patient to a psychiatrist or a psychologist and decline the procedure	24 (11.3%)
First, I refer the patient to a psychiatrist or a psychologist and possibly carry out the requested procedure later	25 (11.7%)
I carry out the procedure in parallel with psychological care	05 (02.3%)
Have you ever refused to treat a patient with (the suspicion of) BDD?	
I’ve never dealt with this	12 (05.6%)
I would do so if this happened	79 (37.1%)
I have done so with a number of patients	97 (45.5%)
This is what I always do	25 (11.7%)
Have you ever been threatened by a patient with BDD	
No, this has never happened to me	32 (15.0%)
I have been physically threatened	82 (38.5%)
I have been verbally threatened	82 (38.5%)
I have been threatened with legal steps	17 (08.0%)

Significant relations

Table 4 shows a significant correlation between BDD knowledge and certain socio-demographic factors. younger age (p = 0.020), employment in a private clinic (p = 0.029), and a lower annual patient influx (p = 0.014) were associated with greater knowledge about BDD. Surgeons in general hospitals were less knowledgeable about BDD (p = 0.050).

**Table 4:** Relationship between having knowledge about BDD and the socio-demographic characteristics of plastic surgeons (n = 213) † Some surgeons have more than one employment setting. χ^2^: Chi-square test § p-value has been calculated using the chi-square test. ** Significant at p < 0.05 BDD, body dysmorphic disorder

Factor	Knowledge about BDD		χ^2^	p-value ^§^
	Yes, n (%) ^(n = 200)^	No, n (%) ^(n = 13)^		
Age group				
≤40 years	97 (48.5%)	02 (15.4%)	5.381	0.020 **
>40 years	103 (51.5%)	11 (84.6%)		
Gender				
Male	104 (52.0%)	08 (61.5%)	0.445	0.505
Female	96 (48.0%)	05 (38.5%)		
Nationality				
Saudi	166 (83.0%)	10 (76.9%)	0314	0.575
Non-Saudi	34 (17.0%)	03 (23.1%)		
Years of experience				
≤10 years	67 (33.5%)	01 (07.7%)	3.741	0.053
>10 years	133 (66.5%)	12 (92.3%)		
Current position				
Consultant	104 (52.0%)	07 (53.8%)	0.017	0.897
Specialist/physician	96 (48.0%)	06 (46.2%)		
Employment setting ^†^				
General hospital	114 (57.0%)	11 (84.6%)	3.839	0.050 **
Private clinic	136 (68.0%)	05 (38.5%)	4.760	0.029 **
Self-employed	35 (17.5%)	02 (15.4%)	0.038	0.845
University hospital	28 (14.0%)	01 (07.7%)	0.413	0.520
Number of new patients each year				
≤350	65 (32.5%)	0	6.081	0.014 **
>350	135 (67.5%)	13 (100%)		
Level of attitude toward BDD				
Negative	17 (08.5%)	02 (15.4%)	0.718	0.698
Neutral	148 (74.0%)	09 (69.2%)		
Positive	35 (17.5%)	02 (15.4%)		

Lastly, as presented in Table 5, higher attitude scores were associated with older age (Z = 3.336; p = 0.001) and employment in a university hospital (Z = 2.068; p = 0.039). No significant relationships were found between attitude scores and sex, years of experience, current position, or annual patient numbers (p > 0.05).

**Table 5:** Differences in the score of attitude in relation to the socio-demographic characteristics of plastic surgeons (n = 213) † Some surgeons have more than one employment setting. § p-value has been calculated using Mann Whitney Z-test. ** Significant at p < 0.05

Factor	Attitude score (60), mean ± SD	Z-test	p-value ^§^
Age group			
≤40 years	38.0 ± 6.19	3.336	0.001 **
>40 years	40.8 ± 8.38		
Gender			
Male	39.1 ± 7.21	0.484	0.629
Female	39.9 ± 7.93		
Nationality			
Saudi	38.9 ± 6.65	2.562	0.010 **
Non-Saudi	42.6 ± 10.5		
Years of experience			
≤10 years	38.3 ± 4.99	1.668	0.095
>10 years	40.1 ± 8.46		
Current position			
Consultant	39.3 ± 7.30	0.221	0.825
Specialist/physician	39.7 ± 7.86		
Employment setting ^†^			
General hospital	39.3 ± 6.81	0.655	0.513
Private clinic	38.8 ± 6.89	1.220	0.222
Self-employed	40.4 ± 8.00	0.899	0.369
University hospital	42.1 ± 7.62	2.068	0.039 **
Number of new patients each year			
≤350	38.1 ± 5.68	1.735	0.083
>350	40.1 ± 8.19		

## Discussion

This study evaluated the knowledge and attitudes toward BDD among plastic surgeons in Saudi Arabia. Consistent with other studies [7,10,12-14], our surgeons were familiar with BDD; only 7% (n = 15) of participants were unfamiliar with BDD’s clinical manifestations. Regarding their knowledge sources, most of our study’s participants indicated they understood BDD through literature reviews, lectures, and conferences. This pattern aligns with a previous study conducted in Saudi Arabia [12]. Conversely, in Bouman et al.’s study [10], the participants were members of a Dutch professional association for aesthetic plastic surgery, dermatology, and cosmetic medicine who acquired their knowledge primarily from conferences and lectures, relegating general literature to a secondary role.

Additionally, many cosmetic surgeons reported encountering patients with BDD in the past year, aligning with findings from other studies [10,12]. The symptoms most frequently observed among the study’s participants included "dissatisfaction with previous cosmetic surgery," reported by 68.5% (n = 146). This contrasts with Kattan’s study [12], where 62.6% noted "excessive concern with, or distress over, minor or nonexistent appearance flaws" as the most common symptom. This disparity may be attributed to the fact that all participants in the current study were plastic surgeons, as opposed to 36.1% in the local study, which included plastic surgeons, dermatologists, and otolaryngologists [12]. A similar study among dermatological surgeons in the United States showed that 93% of participants reported excessive concern or distress over minor or nonexistent appearance features as the most prevalent symptom [13].

Moreover, most participants in the current study tended to openly discuss BDD when interacting with patients. Only 5.6% (n = 12) refrained from addressing the potential presence of BDD. This is in accord with other international studies [10,13]. However, this contrasts with one local study where 30% of participants avoided addressing the potential presence of BDD. Most participants deemed BDD a definitive reason to abstain from aesthetic procedures, with only 0.5% considering it not a contraindication. In Kattan’s study, 10.5% considered BDD a contraindication for aesthetic surgery [12]. Nearly half (46.2%) of our participants involved psychological support services when treating such individuals, echoing findings in other studies [10,13].

Regarding potential aggression, 85% (n = 164) of our plastic surgeon participants experienced threats from BDD patients. A smaller fraction, 8% (n = 17), faced legal threats, and 38.5% (n = 82) encountered physical threats, a pattern not corroborated by other international or local studies [10,12,13]. A 2002 study of plastic surgeons estimated that 40% had received threats from BDD patients [7]. In contrast, an earlier local study reported that 83% of respondents had not received threats [12], and an international study indicated this figure at 77% [10]. In an American study among dermatologic surgeons, only 1% encountered any form of threat, either legal or physical [13]. The latter two studies predominantly focused on dermatologists, possibly explaining the differing rates of reported threats and underscoring the risks of surgeries on individuals with BDD diagnoses.

Lastly, the limitations of our study include reliance on self-assessment rather than direct observation, which may lead to recall bias, and using a convenience sampling technique. Around 50% of the sample size responded to the e-mail survey, raising concerns about the attitudes of non-responders.

## Conclusions

In conclusion, few studies have explored the awareness of plastic surgeons regarding BDD in Saudi Arabia. This study shows that plastic surgeons are highly familiar with BDD. Notably, a small proportion of participants viewed aesthetic procedures as part of psychotherapy, emphasizing the need for further research. Additionally, investigating the influence of social and cultural factors on Saudi plastic surgeons' attitudes is crucial. The findings of this study could be valuable in shaping policies and providing recommendations, including screening tools to assist plastic surgeons and cosmetic treatment institutes in managing BDD patients. Unlike other research in this area, a significant proportion of our participants reported receiving threats from BDD patients, highlighting the need to explore the risk of post-surgery violence in individuals with BDD.

## Appendices

**Table 6:** Survey

Demographic Data					
1- Age:					
2- Gender: a. male b. female					
3- Nationality					
Ο Saudi					
Ο Non-Saudi					
4- Years of experience:					
Ο Less than 5 years					
Ο 5-10					
Ο 10-15					
Ο 15-20					
5- current position:					
consultant					
specialist					
board certified physician					
6- Employment setting:( you can choose multiple answers)					
Ο General hospital					
Ο Private clinic					
Ο Self-employed					
Ο University hospital					
7- Number of new patients each year					
Ο Less than 200					
Ο 200-350					
Ο 350-500					
Ο More than 500					
Familiarity with Body Dysmorphic Disorder					
1. The following is a list of Body Dysmorphic Disorder symptoms, please select the symptoms that you have most frequently encountered/ seen in patients of whom you suspected to have BDD (check all that apply)					
a. Excessive concern with, or distress over, minor or nonexistent appearance flaws					
b. Dissatisfaction with previous cosmetic surgery					
c. Unusual or excessive requests for cosmetic surgery					
d. References to others taking special note of the perceived appearance flaw					
e. Belief that the procedure will transform patient’s life or solve all problems					
2. Are you familiar with the diagnostic criteria for BDD?					
f. I’m seeing these criteria now for the first time					
g. I’ve heard of these					
h. I’m slightly familiar with these					
i. I have been familiar with these for a long time					
3. Are you familiar with the clinical picture of BDD?					
a. I’m not familiar with this					
b. I’m partly familiar with this					
c. I’m reasonably familiar with this					
d. I’m totally familiar with this					
4. How did you acquire knowledge on BDD?					
a. No knowledge					
b. General professional literature					
c. Specific literature on BDD					
d. Conferences or lectures					
e. Colleagues					
f. Web sites					
5. Did you encounter patients with BDD over the past year?					
a. No, I didn’t encounter any					
b. Yes, by hindsight I’ve probably encountered them					
c. Yes, by hindsight I’ve certainly encountered them					
d. Yes, I’ve certainly encountered them					
6. How many patients with BDD did you see last year?					
a. None					
b. 1–5 patients					
c. 5–10 patients					
d. >11 patients					
Attitudes Toward Body Dysmorphic Disorder and Cosmetic Surgery					
In this section, there are 12 statements of job-related feelings. Please read each statement carefully and decide if you agree, disagree, or neutral from 1-5					
	1	2	3	4	5
Statement	Don’t agree at all		neutral		Totally agree
1. I usually get a gut feeling that something is wrong when seeing patients with BDD					
2.I find it challenging to deal with patients with BDD					
3. I find BDD a contraindication for an aesthetic procedure					
4. Patients with BDD have realistic expectations on the impact of the aesthetic procedure on their daily functioning					
5. Patients with BDD have realistic expectations on the physical results of the aesthetic procedure					
6. Patients with BDD benefit equally from aesthetic procedures as other patients					
7. If a patient wants an aesthetic procedure, I will always carry this out					
8. If I think an aesthetic procedure to be unnecessary, I’ll tell the patient					
9. Even if I find an aesthetic procedure unnecessary, I’ll still care this out					
10. If I find an aesthetic procedure unnecessary, I refer the patient to a psychiatrist/psychologist					
11. Aesthetic procedures are a luxury article, but also patient care					
12. Aesthetic procedures are basically a kind of “psychotherapy/psychosurgery					
Experience with Body Dysmorphic Disorder					
1. Do you explore BDD or disturbed body image during the intake interview?					
a. Never					
b. Sometimes					
c. Often					
d. Always					
2. In patients with BDD, do you shift the topic from the technical aspects of the procedure to body image problems?					
a. No. Body image is never part of the intake interview					
b. Sometimes					
c. Most of the time					
d. Always, because body image is a standard topic during the intake interview					
3. In case of BDD or disturbed body image, do you share this with your patient?					
a. No, I keep that to myself					
b. Sometimes					
c. Most of the time					
d. Yes, always					
4. What do you do when you recognize or suspect BDD in a patient?					
a. I don’t address this					
b. I approach such a patient no different from other patients					
c. I share my impression on this patient’s appearance					
d. I talk about the patient’s disturbed body image					
e. First, I consult a psychiatrist/psychologist about what to do					
f. I refer the patient to a psychiatrist or a psychologist and decline the procedure					
g. First, I refer the patient to a psychiatrist or a psychologist, and possibly carry out the requested procedure later					
h. I carry out the procedure in parallel with psychological care					
5. Have you ever refused treating a patient with (the suspicion of) BDD?					
a. I’ve never dealt with this					
b. b. I would do so, if this would happen					
c. c. I have done so in a number of patients					
d. This is what I always do					
6. Have you ever been threatened by a patient with BDD?					
a. No, this has never happened to me					
b. I have been physically threatened					
c. I have been verbally threatened					
d. I have been threatened with legal steps					

## References

[1] American Psychiatric Association (2022). Obsessive-compulsive and related disorders. Diagnostic and Statistical Manual of Mental Disorders (5th Edition).

[2] IsHak WW, Bolton MA, Bensoussan JC (2012). Quality of life in body dysmorphic disorder. CNS Spectr.

[3] Phillips KA, Dufresne RG (2000). Body dysmorphic disorder. A guide for dermatologists and cosmetic surgeons. Am J Clin Dermatol.

[4] Shaffi Ahamed S, Enani J, Alfaraidi L, Sannari L, Algain R, Alsawah Z, Al Hazmi A (2016). Prevalence of body dysmorphic disorder and its association with body features in female medical students. Iran J Psychiatry Behav Sci.

[5] Mortada H, Seraj H, Bokhari A (2020). Screening for body dysmorphic disorder among patients pursuing cosmetic surgeries in Saudi Arabia. Saudi Med J.

[6] Phillips KA, Grant J, Siniscalchi J, Albertini RS (2001). Surgical and nonpsychiatric medical treatment of patients with body dysmorphic disorder. Psychosomatics.

[7] Sarwer DB, Crerand CE (2002). Psychological issues in patient outcomes. Facial Plast Surg.

[8] Crerand CE, Phillips KA, Menard W, Fay C (2005). Nonpsychiatric medical treatment of body dysmorphic disorder. Psychosomatics.

[9] Khattab NR, Mills D (2021). BDD knowledge, attitude and practice among aesthetic plastic surgeons worldwide. Aesthetic Plast Surg.

[10] Bouman TK, Mulkens S, van der Lei B (2017). Cosmetic professionals’ awareness of body dysmorphic disorder. Plast Reconstr Surg.

[11] Sarwer DB (2002). Awareness and identification of body dysmorphic disorder by aesthetic surgeons: results of a survey of American Society for Aesthetic Plastic Surgery members. Aesthet Surg J.

[12] Kattan AE, Alnujaim NH, Barasain O, Bouman TK, AlHammad R, Van der Lei B (2020). Awareness and experiences of cosmetic treatment providers with body dysmorphic disorder in Saudi Arabia. PeerJ.

[13] Sarwer DB, Spitzer JC, Sobanko JF, Beer KR (2015). Identification and management of mental health issues by dermatologic surgeons: a survey of American Society for Dermatologic Surgery members. Dermatol Surg.

[14] Szepietowski JC, Salomon J, Pacan P, Hrehorów E, Zalewska A (2008). Body dysmorphic disorder and dermatologists. J Eur Acad Dermatol Venereol.

